# Supplementary material for: Carbon pricing and planetary boundaries
Source: Nat Commun. 2020 Sep 17;11:4688. doi: 10.1038/s41467-020-18342-7 (PMC7498463; doi:10.1038/s41467-020-18342-7)
Supplement: Supplementary file 1 — Supplementary Information [file 41467_2020_18342_MOESM1_ESM.pdf]

Supplementary Information:
Carbon pricing and Planetary Boundaries

Engström et.al.

Supplementary Note 1

Drivers of planetary pressures

We identify the principal sources of anthropogenic pressure on each Earth system process (ESP). The results are summarized in Supplementary Table 1 and detailed below.

Climate change is caused by an increased atmospheric concentration of greenhouse gases, and anthropogenic CO_2 emissions have been identified, with a high degree of confidence, as the main driver of this increase [1]. The planetary boundary is set by [2] to a CO_2 concentration of 350 parts per million. The largest source of anthropogenic CO_2 emissions is fossil fuel consumption, primarily from energy production (approximately 66.5% of total emissions), which includes electricity and heat (24.9%), industry (14.7%) and transportation (14.3%). Agriculture is the second largest contributor (13.8%), mainly through livestock production. The third largest contributor is land-use change (12.2%), mainly through deforestation [3]. More details on how emissions are related to specific variables in our model can be found in the Methods section. In total the included model variables cover approximately 97% of total emissions.

Biogeochemical flows of phosphorus and nitrogen from soils into freshwater systems and oceans can cause widespread eutrophication and large-scale anoxic events. Steffen et al. [2] sets the boundary to application of no more than 6.2 Teragram (Tg) of phosphorus per year to erodible soils, and no more than 62 Tg of nitrogen. Nearly all excess phosphorus and nitrogen loading comes from the agricultural sector. Almost 90% of the global phosphate rock production is used to make fertilizer [4], and in many regions and watersheds, runoff created by applying synthetic nitrogen fertilizer in agricultural production dominates the nitrogen flux [5]. These fertilizers have been instrumental in achieving rapid agricultural productivity growth in the past half-century [6], but as a result the global surplus of phosphorus increased from 2 Tg to 11 Tg per year to between 1950 and 2000, and nitrogen increased from 36 Tg to 138 Tg

per year [7]. We also note that the industrial process for fixing Nitrogen from the air is heavily reliant on natural gas, which accounts for 72-85% of the production cost [8].

Ocean acidification is in large part caused by an increase in the concentration of free H^+ ions in the surface ocean, which makes it harder to synthesize the aragonite that makes up shells and corals. The boundary set by Steffen et al. [2] is maintaining at least 80% of the pre-industrial aragonite saturation state to prevent serious deterioration. The increase in the concentration of free H^+ ions occurs primarily as a consequence of an increased atmospheric CO_2 concentration, and in keeping this below 350 parts per million we would also stay on the right side of the ocean acidity boundary. It is therefore mainly fossil fuel-based energy production and agricultural emissions that create pressure on this planetary process. But agricultural production also contributes to ocean acidification by a different mechanism. Nutrient runoff can fuel massive algal blooms, which deplete bottom waters of oxygen and release CO_2 when the organic matter from these blooms is respired by bacteria [9]. In our impact analysis, however, we do not consider this additional effect but instead follow [2] and consider only the dominant driver i.e. atmospheric carbon dioxide concentration.

Freshwater use in excess of net natural recharge can lead to regime shifts in ecosystems that depend on flows from rivers, lakes, and renewable groundwater stores. Preventing this requires management at the basin-level, but taken in aggregate, prudent management requires global consumption to remain below 4,000 cubic kilometers of freshwater per year [2]. Agriculture is the largest consumptive user of freshwater, accounting for an estimated 92% of global freshwater use annually [10].

Land-use change, specifically the conversion of forested land to other uses, can substantially affect the climate by altering evapo-transpiration and the albedo of the land surface. Steffen et al. [2] set the boundary at maintaining at least 75% of original forest cover, to avoid such disruptive changes. Agriculture is today the largest user of land on the planet (about two fifths)

[11, 12], and the main driver of land use change. Between 1980 and 2000, more than 55% of new agricultural land across the tropics came at the expense of intact forests, and another 28% came from disturbed forests [13]. Agricultural expansion is, by far, the leading land-use change associated with nearly all deforestation cases (96%) [14].

Biodiversity loss undermines functional diversity and can lead to persistent loss of ecosystem productivity. Steffen et al. [2] suggest that we should aspire to cause no more than one extinction per million species-years (the background extinction rate), but that the planetary boundary is at about 10 extinctions per million species-years, where one extinction per million species-years would imply that, if there are a million species on earth, one would go extinct every year, on average. Due to the difficulty in finding data on extinction rates we instead rely on data on number of threatened species from the International Union for Conservation of Nature (IUCN) red list. Maxwell et al. [15] recently analysed 8,688 threatened or near-threatened species from the IUCN red list, and concluded that crop farming poses the single greatest threat. Agricultural activity negatively affected 62% of those listed as threatened or near-threatened. Likewise, 46% of the total number of species was also threatened by logging activities. However, biodiversity loss is also an issue in marine environments, impairing the ocean's capacity to provide food, maintain water quality, and recover from perturbations [16]. Global fish catches have increased 80 fold in volume since 1950, reaching around 144 million tonnes in 2006 [17]. Evidence of adverse human influence can be found in essentially all marine ecosystems [18].

Atmospheric aerosol loading impacts on climate change by both scattering and absorbing solar and infrared radiation in the atmosphere, but also indirectly by, e.g., altering the precipitation efficiency of clouds [19]. Aerosols also has serious impacts on human health leading to several millions of human casualties per year [20]. Steffen et.al. [2] adopt aersosol optical depth (AOD) as a control variable but do not provide any quantitative assessment of a global boundary. Here, we also adopt aerosol optical depth (AOD) as a measure of atmospheric aerosol

loading. AOD is affected by aerosol emissions from black carbon, organic carbon, sulfates, nitrates, sea salt and mineral dust originating from both natural causes, e.g. forest and grassland fires, but also man-made contributions from industrial and agricultural activity. We base our measure of AOD on the primary aerosol components sulfate, black carbon, and organic carbon, identified in Streets et.al. [21]. Together with emission data from [22] we were able to back out a link between our model variables fossil fuel, biofuel and land-use change (a major driver of biomass burning) and these three source components.

Ozone depletion, discovered in 1982, is mainly caused by anthropogenic emissions of ozone-depleting substances. Most of these emissions have been successfully controlled by the Montreal protocol and the ozone layer has started to recover. Given the reductions of these emissions, nitrous oxide N_2O is now (and, if left unregulated, is expected to be throughout the 21st century) the most important ozone depleting substance [23]. Based on table 7.7 in [24], out of anthropogenic emissions, agriculture account for 42%, fossil fuel combustion and industrial processes for 10%, and biomass and biofuel burning for 10%.

In addition to that, climate change affects ozone depletion. Climate change will increase the tropospheric temperature but decrease stratospheric temperature. This will lead to decreased ozone depletion in some places and increased ozone depletion in others, particularly in the arctic [25].

Chemical pollution has, in the updated version of the Planetary Boundaries [2], been widened to the new category of novel entities. The following discussion is based on the original category chemical pollution as described in [20].

This is still a very broad category containing a large and heterogenous set of substances. This makes it difficult to construct a combined measure of the global amounts and define an associated planetary boundary. In all [26, 20, 2] the boundary is left unspecified. We will, however, argue that the emissions of most of these substances can be relatively closely linked

to variables in our model and hence that we can say something about whether they will increase or decrease in response to our considered policies. For instance, we will link chemicals emitted in industrial processes or by industrially produced household chemicals to our model variable manufacturing and we will link the use of pesticides to our model variable total agricultural production.

Based on (https://www.environmentalpollutioncenters.org/chemical/causes/) we construct the following list of problematic chemical pollutants still used and their sources.

Organic chemical pollutants:

- **Crude oil and petroleum refined products** linked to our variable fossil fuel extraction.
- **Solvents** (e.g., acetone, MEK, toluene, benzene, xylene) used in industry as well as in many household products; linked to our variable manufacturing.
- **Chlorinated solvents** (e.g., PCE, TCE, 1,1,1-TCA, 1,2-DCA, 1,1,2-TCA) used in industrial degreasing processes, as well as in dry cleaning, and in various household products; linked to our variabel manufacturing.
- **PAHs (polyaromatic hydrocarbons)** are found in petroleum products, crude oil, but are also a result of burning activities (e.g., from coal power plants as well as historical manufacturing gas plants); linked to our variable extracted fossil fuels.
- **Alcohols (e.g., ethanol, methanol, isopropanol)** are used in a large variety of applications and household products; difficult to link to specific model variable.
- **Trihalomethanes** (e.g., chloroform, dibromochloromethane, chlorobromomethane, bromoform) which are common products of water chlorination; difficult to link to specific model variable.

- **Phenols** are usually an indication of waste water and a result of industrial processes; linked to our variable manufacturing.
- **Plastics** are a result of industrial processes as well as our daily activities involving using and disposing of a large variety of plastics (e.g., bags, bottles, containers); linked to our variable manufacturing.
- **Pesticides / Insecticides / Herbicides** are commonly used in agriculture and may contain toxic organic chemicals and metals (such as mercury and arsenic); linked to our variable total agricultural production.
- **Detergents** (e.g., nonylphenol ethoxylate) include a variety of chemical compounds with surface activity; difficult to link to specific model variable.
- **Organo-metallic compounds** (e.g., organo-arsenicals, organo-mercurials) are usually pesticides / insecticides / herbicides; linked to our variable total agricultural production.

Inorganic chemical pollutants:

- **Metals and their salts** – usually from mining and smelting activities, as well as disposal of mining wastes; difficult to link to specific model variable.
- **Inorganic fertilizers** (e.g., nitrates, phosphates) used largely in agriculture and gardening;linked to our variable fertilizers.
- **Sulfides** (such as pyrite) are usually mined minerals and once disposed of in the environment, they may generate sulfuric acid in the presence of precipitation water and microorganisms; difficult to link to specific model variable.

- **Ammonia** is released mainly by fertilizer use, livestock, industry and transport [27]; linked to our variables fossil fuel use in fertilizer production (proxy for nitrogen part of fertilizers), agricultural production for food and manufacturing.

- **The oxides of nitrogen and sulfur** are very common air pollutants resulting from vehicle emissions, industrial processes, and other human activities; linked to our variables fossil fuel use in energy services production and manufacturing.

- **Acids and bases** are used in a variety of industrial applications as well as in chemical laboratories. These are less problematic chemicals because their effect can be easily neutralized in the environment, but if spread in large amounts they may still pose a threat to environment and human health; linked to our variable manufacturing.

- **Perchlorate** includes the perchloric acids and its various salts. Perchlorate is used in a variety of applications including rocket fuel, explosives, military operations, fireworks, road flares, inflation bags, etc. Perchlorate is problematic because it is persistent and may damage thyroid function in humans; difficult to link to specific model variable.

While some substances where difficult to pin to specific model variables, we would assume most of them do increase with increased economic activity as captured by our variable manufacturing.

Finally, we assume that chemical pollution is increasing in manufacturing, extracted fossil fuels, total agricultural production, agricultural production for food, fossil fuel use in fertilizer production and fossil fuel use in energy services production. Determining the quantitative response of a given chemical to model variables is beyond the scope of this analysis.

To summarize, the key economic drivers of planetary pressures appear to be fossil fuel consumption and agricultural production, and to a lesser extent logging, fisheries and manufacturing. Fossil fuel consumption is clearly a key driver of climate change and ocean acidification,

and is associated more indirectly with other ESPs through economic linkages. Fossil fuel consumption together with biofuels is also a primary driver of aerosol loading. Although agriculture only accounts for about 4% of global economic output [28], it has an outsized impact on many ESPs. It uses nearly 40% of the Earth's land surface, contributes 24% of global greenhouse gas emissions, accounts for over 90% of global freshwater use, Phosphorus use, and Nitrogen use. Both fossil fuel consumption and agriculture, as well as their interaction, will therefore have to be central features of any model that aims to capture how human activity puts pressure on the planetary boundaries. More details on the central role of agriculture as driver of planetary pressures can be found in [29].

Supplementary Note 2

Elasticity of emissions to the carbon price

When considering a carbon price as the only policy, a one percentage point increase in the carbon tax leads to a 0.36% decrease of emissions. We will here compare this central result to other model based and empirical results. We start with results from other models.

From [30] we can back out the initial effect on emissions of a carbon tax. There are two types of fossil fuel in the model: coal and oil. With a homogenous price on emissions (in $ per ton of carbon) the percentage tax rate differs between the sources. On page 69 we find that the fuel price, expressed as $ per ton of carbon, is 103.65 for coal and 606.5 for oil. On page 70 the tax is said to be 56.6 $ per ton of carbon. Combining these numbers, we find that the tax rate is 54% for coal and 9.4% for oil. On page 72 it says that the initial decrease in coal use in response to the tax is 46%. For oil it says on page that oil use in all periods is changed by at most 6% and in the graph it seems that the change is the largest initially at seems to be about 6%. We thus use an initial decrease in oil use of 6%. Together these numbers imply that that the change in emissions per percentage point carbon tax is about 0.85% for coal and 0.64% for

oil.

Another model based estimate of the sensitivity comes from [31], based on the analysis in [32]. They find that for the US (based on an average over 11 included models) the effect of a carbon tax would be to decrease emissions by between 1.11% per percentage point of tax (based on a tax of \$25 per ton of CO_2) and 0.67% per percentage point of tax (based on a tax of \$50 per ton of CO_2).

Hence, we can see that compared to these models, emissions in our model are less sensitive to a carbon price.

When it comes to empirical values, there are, for obvious reasons, no estimates of the effects of a global carbon price. Many studies find small, imprecisely estimated or insignificant effects of carbon taxes (see [31] for examples). There are, however, a number of methodological challenges involved. [33] analyze the effects of the Swedish fuel tax using a synthetic control method. We will here consider the effect of the tax during the period 1990-2000. Going to the data sources for figure 1, the average carbon tax for that period is 0.766 SEK/litre. In the analysis the VAT that is applied to the after tax price is counted as part of the carbon tax. The VAT rate is 25% and hence the average carbon tax including VAT is 0.957 SEK/litre. The average price at the pump (including all taxes) was 7.18 SEK/litre. The percentage that the carbon tax (including VAT) adds to the price is thus

$$\tau_E = \frac{7.18}{7.18 - 0.957} - 1 \approx 15.4\%.$$

[33] finds (on page 23) that the average decrease in gasoline use attributable to the carbon tax was 4.8%. The change in gasoline use per percentage point of tax is thus about 0.31. This study considers a small open economy where supply of fossil fuel (in this case gasoline) can be expected to be completely elastic. This is also confirmed by the finding that there is more or less complete pass through of the tax to consumer prices. In our setting, there is a reaction in

the (pre-tax) fossil fuel price that further limits the effects of the price. With such an effect the elasticity in [33] would likely be smaller.

Summing up, the elasticity of emissions from fossil fuels with respect to a carbon tax is lower than in some other models, but seems relatively well inline with empirical estimates.

Supplementary Methods

Deriving equilibrium and comparative-statics conditions

We will here provide a brief description of how the equilibrium conditions are derived and how they, in turn, can be used to derive the comparative statics conditions. This will also illustrate the entities for which we need empirical estimates to parameterize the model. As an example, we can consider the industrial-manufacturing sector. The first-order conditions associated with maximization problem (5) are

$$\frac{p_{\mathcal{E}}}{p_Y} - \frac{\partial Y}{\partial \mathcal{E}_Y} = 0 \text{ and } \frac{p_{M_Y}}{p_Y} - \frac{\partial Y}{\partial M_Y} = 0. \tag{S.1}$$

These are examples of the G_j in (14). As described above, the complete set of equilibrium conditions implicitly define all equilibrium prices and quantities as functions of τ. Differentiating the first condition with respect to τ gives

$$\hat{p}_{\mathcal{E}} - \hat{p}_Y - \Gamma^Y_{\mathcal{E}_Y,\mathcal{E}_Y}\hat{\mathcal{E}}_Y - \Gamma^Y_{\mathcal{E}_Y,M_Y}\hat{M}_Y = 0, \tag{S.2}$$

where we have defined

$$\hat{X} \equiv \frac{1}{X}\frac{dX}{d\tau} \text{ and } \Gamma^Z_{X_1,X_2} \equiv \frac{\frac{\partial^2 Z}{\partial X_2 \partial X_1}}{\frac{\partial Z}{\partial X_1}} X_2. \tag{S.3}$$

For the fossil fuel extraction sector, the first order condition of the profit maximization problem (7) is

$$\frac{p_E}{1+\tau_E} - g'_E(E) = 0. \tag{S.4}$$

When differentiating with respect to τ_E, we need to consider the direct effect in addition to the induced changes in p_E and E. The comparative statics condition is

$$\hat{p}_E - \Lambda_E \hat{E} = \frac{1}{1+\tau_E}, \tag{S.5}$$

where

$$\Lambda_X \equiv \frac{X g_X''(X)}{g_X'(X)} \tag{S.6}$$

is the inverse of the supply elasticity of X. For the sectors described in (8), the computations are the same except that there is no direct effect of the tax.

Finally, differentiating the market-clearing conditions, here exemplified by the condition for land (10), with respect to τ_E gives

$$Q_{L,L_A}\hat{L}_A + Q_{L,L_T}\hat{L}_T + Q_{L,L_U}\hat{L}_U = 0, \tag{S.7}$$

where

$$Q_{X,X_Z} \equiv \frac{X_Z}{X} \tag{S.8}$$

is the share of good X used in sector Z (or for consumption if $Z = U$).

From the derived comparative statics conditions, we can identify the things that we need empirical values for in order to compute the effects of the tax. The last condition contains the quantity shares going to the different land uses. Similarly, we will need quantity shares for all the market-clearing conditions (11)-(13). We will also need the supply elasticities (S.6). Finally, we will need the factors defined in (S.3) for the production and utility functions. As we will show below, the empirical values needed for these are, for our chosen functional forms, elasticites of substitution and expenditure shares (i.e. shares of total expenditures spend on a given input or consumption good).

Biofuel policy

The tax on biofuels can be implemented by replacing the maximization problem for the producer of energy services (2) by the problem

$$\max_{A_B, E_\mathcal{E}, R} p_\mathcal{E}\mathcal{E}(A_B, E_\mathcal{E}, R) - p_A(1+\tau_B)A_B - p_E E_\mathcal{E} - p_R R.$$

Functional forms

First we will define another expression for the utility or production function Z:

$$\Gamma_X^Z \equiv \frac{X}{Z}\frac{\partial Z}{\partial X}. \tag{S.9}$$

This is the output elasticity of Z with respect to X. Empirically it is also the share of total spending that is spent on X, we will refer to this quantity as the expenditure share from here on:

$$\Gamma_X^Z = \frac{p_X X}{p_Z Z}. \tag{S.10}$$

For industrial manufacturing, fisheries, timber, fertilizer production and energy services we assume one-level Constant Elasticity of Substitution (CES) functions. More precisely the production functions are

$$Y = \left[\gamma_{Y,M_Y} M_Y^{\frac{\sigma_Y - 1}{\sigma_Y}} + \gamma_{Y,\mathcal{E}_Y}\mathcal{E}_Y^{\frac{\sigma_Y - 1}{\sigma_Y}}\right]^{\frac{\sigma_Y}{\sigma_Y - 1}} \tag{S.11}$$

for industrial manufacturing,

$$F = \left[\gamma_{F,M_F} M_F^{\frac{\sigma_F - 1}{\sigma_F}} + \gamma_{Y,E_F} E_F^{\frac{\sigma_F - 1}{\sigma_F}}\right]^{\frac{\sigma_F}{\sigma_F - 1}} \tag{S.12}$$

for fisheries,

$$T = \left[\gamma_{T,M_T} M_T^{\frac{\sigma_T - 1}{\sigma_T}} + \gamma_{T,L_T} L_T^{\frac{\sigma_T - 1}{\sigma_T}}\right]^{\frac{\sigma_T}{\sigma_T - 1}} \tag{S.13}$$

for timber,

$$P = \left[\gamma_{P,E_P} E_P^{\frac{\sigma_P - 1}{\sigma_P}} + \gamma_{P,\mathcal{P}}\mathcal{P}^{\frac{\sigma_P - 1}{\sigma_P}} + \gamma_{P,M_P} M_P^{\frac{\sigma_P - 1}{\sigma_P}}\right]^{\frac{\sigma_P}{\sigma_P - 1}} \tag{S.14}$$

for fertilizers and

$$\mathcal{E} = \left[\gamma_{\mathcal{E},A_B} A_B^{\frac{\sigma_{\mathcal{E}}-1}{\sigma_{\mathcal{E}}}} + \gamma_{\mathcal{E},E_{\mathcal{E}}} E_{\mathcal{E}}^{\frac{\sigma_{\mathcal{E}}-1}{\sigma_{\mathcal{E}}}} + \gamma_{\mathcal{E},R} R^{\frac{\sigma_{\mathcal{E}}-1}{\sigma_{\mathcal{E}}}}\right]^{\frac{\sigma_{\mathcal{E}}}{\sigma_{\mathcal{E}}-1}} \tag{S.15}$$

for energy-services production.

For CES function

$$Z = \left[\sum_i \gamma_{Z,X_i} X_i^{\frac{\sigma_Z-1}{\sigma_Z}}\right]^{\frac{\sigma_Z}{\sigma_Z-1}},$$

(S.3) becomes

$$\Gamma^Z_{X_i,X_k} = \begin{cases} -\frac{1}{\sigma_Z}\left[1-\Gamma^Z_{X_i}\right] & \text{if } k=i \\ \frac{1}{\sigma_Z}\Gamma^Z_{X_k} & \text{if } k\neq i \end{cases}. \tag{S.16}$$

Hence they can be expressed completely in terms of σ_Z and the expenditure shares Γ^Z_X.

For agricultural production we assume a two-level CES production function

$$A(\tilde{L}_A, L_A) = \left[\gamma_{A,\tilde{L}_A} \tilde{L}_A^{\frac{\sigma_A-1}{\sigma_A}} + \gamma_{A,L_A} L_A^{\frac{\sigma_A-1}{\sigma_A}}\right]^{\frac{\sigma_A}{\sigma_A-1}},$$

where

$$\tilde{L}_A = \left[\gamma_{\tilde{L}_A,M_A} M_A^{\frac{\sigma_{\tilde{L}_A}-1}{\sigma_{\tilde{L}_A}}} + \gamma_{\tilde{L}_A,P} P^{\frac{\sigma_{\tilde{L}_A}-1}{\sigma_{\tilde{L}_A}}} + \gamma_{\tilde{L}_A,W} W^{\frac{\sigma_{\tilde{L}_A}-1}{\sigma_{\tilde{L}_A}}} + \gamma_{\tilde{L}_A,\mathcal{E}_A} \mathcal{E}_A^{\frac{\sigma_{\tilde{L}_A}-1}{\sigma_{\tilde{L}_A}}}\right]^{\frac{\sigma_{\tilde{L}_A}}{\sigma_{\tilde{L}_A}-1}}.$$

Finally, the households' utility function is a two-level CES function

$$U(\tilde{\mathcal{F}}, \mathcal{F}) = \left[\gamma_{U,\tilde{\mathcal{F}}} \tilde{\mathcal{F}}^{\frac{\sigma_U-1}{\sigma_U}} + \gamma_{U,\mathcal{F}} \mathcal{F}^{\frac{\sigma_U-1}{\sigma_U}}\right]^{\frac{\sigma_U}{\sigma_U-1}},$$

where

$$\mathcal{F} = \left[\gamma_{\mathcal{F},A_{\mathcal{F}}} A_{\mathcal{F}}^{\frac{\sigma_{\mathcal{F}}-1}{\sigma_{\mathcal{F}}}} + \gamma_{\mathcal{F},A_{\mathcal{F}}} F^{\frac{\sigma_{\mathcal{F}}-1}{\sigma_{\mathcal{F}}}}\right]^{\frac{\sigma_{\mathcal{F}}}{\sigma_{\mathcal{F}}-1}} \text{ and}$$

$$\tilde{\mathcal{F}} = \left[\gamma_{\tilde{\mathcal{F}},Y} Y^{\frac{\sigma_{\tilde{\mathcal{F}}}-1}{\sigma_{\tilde{\mathcal{F}}}}} + \gamma_{\tilde{\mathcal{F}},L_U} L_U^{\frac{\sigma_{\tilde{\mathcal{F}}}-1}{\sigma_{\tilde{\mathcal{F}}}}} + \gamma_{\tilde{\mathcal{F}},T} T^{\frac{\sigma_{\tilde{\mathcal{F}}}-1}{\sigma_{\tilde{\mathcal{F}}}}}\right]^{\frac{\sigma_{\tilde{\mathcal{F}}}}{\sigma_{\tilde{\mathcal{F}}}-1}}.$$

For two-level CES functions, the elasticites are

$$\begin{aligned}\Gamma^Z_{X_{1,j},X_{1,j}} =& \frac{1}{\sigma_Z}\left(\Gamma^Z_{X_1}-1\right)\Gamma^{X_1}_{X_{1,j}} + \frac{1}{\sigma_{X_1}}\left(\Gamma^{X_1}_{X_{1,j}}-1\right),\\ \Gamma^Z_{X_{1,i},X_{1,j}} =& \left[\frac{1}{\sigma_Z}\left(\Gamma^Z_{X_1}-1\right)+\frac{1}{\sigma_{X_1}}\right]\Gamma^{X_1}_{X_{1,j}} \text{ for } i\neq j,\\ \Gamma^Z_{X_k,X_{1,j}} =& \frac{1}{\sigma_Z}\Gamma^Z_{X_{1,j}} \text{ for } k\neq 1 \text{ and}\\ \Gamma^Z_{X_{1,j},X_k} =& \frac{1}{\sigma_Z}\Gamma^Z_{X_k} \text{ for } k\neq 1.\end{aligned} \tag{S.17}$$

Again, these are completely determined by elasticities of substitution (σ) and expenditure shares.

Summing up, the empirical values needed to parameterize the model is supply elasticities for goods modeled using a production cost function (S.6); elasticities of substitution (σs) and expenditure shares of inputs (S.10) for production and utility functions; and quantity shares of goods being used in multiple sectors (S.8).

Full set of equilibrium conditions

There are 25 unknown quantities:

A, A_B, $A_{\mathcal{F}}$, E, $E_{\mathcal{E}}$, E_F, E_P, $\mathcal{E}$, $\mathcal{E}_A$, $\mathcal{E}_Y$, F, L_A, L_T, L_U, M_A, M_F, M_P, M_T, M_Y, P, $\mathcal{P}$, R, T, W and Y.

There are also 16 unknown prices:

$p_A, p_E, p_{\mathcal{E}}, p_F, p_L, p_{M_A}, p_{M_F}, p_{M_P}, p_{M_T}, p_{M_Y}, p_P, p_{\mathcal{P}}, p_R, p_T, p_W$ and p_Y.

We thus have a total of 41 unknowns to be pinned down by the equilibrium conditions.

The representative agriculture producer's maximization problem (1) gives first order conditions

$$p_A\frac{\partial A}{\partial L_A} - p_L c_A(L_A) = 0,\; p_A\frac{\partial A}{\partial P} - p_P = 0,\; p_A\frac{\partial A}{\partial M_A} - p_{M_A} = 0,\; p_A\frac{\partial A}{\partial W} - p_W = 0 \text{ and } p_A\frac{\partial A}{\partial \mathcal{E}_A} - p_{\mathcal{E}} = 0. \tag{S.18}$$

Note: Here (and in the timber producer's problem), the clearing cost function is not differentiated with respect to L_A (or L_T) which reflects that the clearing costs are assumed to be marginal

costs that depend on the aggregate clearing.

The representative energy-service producer's maximization problem (2) gives first order conditions

$$p_{\mathcal{E}}\frac{\partial \mathcal{E}}{\partial E_{\mathcal{E}}} - p_E = 0,\ p_{\mathcal{E}}\frac{\partial \mathcal{E}}{\partial A_B} - p_A = 0 \text{ and } p_{\mathcal{E}}\frac{\partial \mathcal{E}}{\partial R} - p_R = 0. \tag{S.19}$$

The representative fertilizer producer's maximization problem (3) gives first order conditions

$$p_P\frac{\partial P}{\partial E_P} - p_E = 0, p_P\frac{\partial P}{\partial \mathcal{P}} - p_{\mathcal{P}} = 0 \text{ and } p_P\frac{\partial P}{\partial M_P} - p_{M_P} = 0. \tag{S.20}$$

The representative timber producer's maximization problem (4) gives first order conditions

$$p_T\frac{\partial T}{\partial L_T} - p_L c_T(L_T) = 0 \text{ and } p_T\frac{\partial T}{\partial M_T} - p_{M_T} = 0. \tag{S.21}$$

The maximization problem of the representative producer in the manufacturing sector is given in (5). The first order conditions are

$$p_Y\frac{\partial Y}{\partial \mathcal{E}_Y} - p_{\mathcal{E}} = 0 \text{ and } p_Y\frac{\partial Y}{\partial M_Y} - p_{M_Y} = 0. \tag{S.22}$$

The first order conditions to the fisheries producer's maximization problem (6) are

$$p_F\frac{\partial F}{\partial E_F} - p_E = 0 \text{ and } p_F\frac{\partial F}{\partial M_F} - p_{M_F} = 0. \tag{S.23}$$

The first order condition of the fossil fuel producer's maximization problem (7) is

$$\frac{p_E}{1+\tau_E} - g_E'(E) = 0. \tag{S.24}$$

The first order conditions of the problems described in (8) are

$$p_X - g_X'(X) = 0 \text{ for } X \in \{\mathcal{P}, W, R, M_A, M_F, M_P, M_T, M_Y\}. \tag{S.25}$$

Finally, the representative household's maximization problem is given in (9). The first order conditions give

$$\frac{p_A}{p_F} - \frac{\frac{\partial U}{\partial A_F}}{\frac{\partial U}{\partial F}} = 0,\ \frac{p_A}{p_{L_U}} - \frac{\frac{\partial U}{\partial A_F}}{\frac{\partial U}{\partial L_U}} = 0,\ \frac{p_A}{p_T} - \frac{\frac{\partial U}{\partial A_F}}{\frac{\partial U}{\partial T}} = 0 \text{ and } \frac{p_A}{p_Y} - \frac{\frac{\partial U}{\partial A_F}}{\frac{\partial U}{\partial Y}} = 0. \tag{S.26}$$

The first order conditions (S.18)-(S.26) in total provide 30 conditions. In addition, there are 4 market clearing conditions (10)- (13). Finally, the budget constraint in (9) and the production functions in the maximization problems (1)- (6) provide the 7 conditions required to pin down all 41 unknown quantities and prices.

Full set of comparative statics equations

We start by differentiating the market clearing conditions:

$$(10) \Rightarrow Q_{L,L_A}\hat{L}_A + Q_{L,L_T}\hat{L}_T + Q_{L,L_U}\hat{L}_U = 0, \tag{S.27}$$

$$(11) \Rightarrow Q_{A,A_B}\hat{A}_B + Q_{A,A_\mathcal{F}}\hat{A}_\mathcal{F} - \hat{A} = 0, \tag{S.28}$$

$$(12) \Rightarrow Q_{E,E_\mathcal{E}}\hat{E}_\mathcal{E} + Q_{E,E_P}\hat{E}_P + Q_{E,E_F}\hat{E}_F - \hat{E} = 0 \text{ and} \tag{S.29}$$

$$(13) \Rightarrow Q_{\mathcal{E},\mathcal{E}_A}\hat{\mathcal{E}}_A + Q_{\mathcal{E},\mathcal{E}_Y}\hat{\mathcal{E}}_Y - \hat{\mathcal{E}} = 0, \tag{S.30}$$

with the quantity shares defined in (S.8). Note that the quantity shares sum to one. In each condition, specifying all shares except one implies a value for the last share. The share computed in this way are Q_{L,L_U}, $Q_{A,A_\mathcal{F}}$, $Q_{A,E_\mathcal{E}}$ and $Q_{\mathcal{E},\mathcal{E}_Y}$.

Similarly, differentiating the production functions gives

$$(1) \Rightarrow \Gamma^A_{L_A}\hat{L}_A + \Gamma^A_{M_A}\hat{M}_A + \Gamma^A_P\hat{P} + \Gamma^A_W\hat{W} + \Gamma^A_{\mathcal{E}_A}\hat{\mathcal{E}}_A - \hat{A} = 0, \tag{S.31}$$

$$(2) \Rightarrow \Gamma^\mathcal{E}_{A_B}\hat{A}_B + \Gamma^\mathcal{E}_{E_\mathcal{E}}\hat{E}_\mathcal{E} + \Gamma^\mathcal{E}_R\hat{R} - \hat{\mathcal{E}} = 0, \tag{S.32}$$

$$(3) \Rightarrow \Gamma^P_{E_P}\hat{E}_P + \Gamma^P_\mathcal{P}\hat{\mathcal{P}} + \Gamma^P_{M_P}\hat{M}_P - \hat{P} = 0, \tag{S.33}$$

$$(4) \Rightarrow \Gamma^T_{L_T}\hat{L}_T + \Gamma^T_{M_T}\hat{M}_T - \hat{T} = 0, \tag{S.34}$$

$$(5) \Rightarrow \Gamma^Y_{\mathcal{E}_Y}\hat{\mathcal{E}}_Y + \Gamma^Y_{M_Y}\hat{M}_Y - \hat{Y} = 0 \text{ and} \tag{S.35}$$

$$(6) \Rightarrow \Gamma^F_{E_F}\hat{E}_F + \Gamma^F_{M_F}\hat{M}_F - \hat{F} = 0, \tag{S.36}$$

with the expenditure shares defined in (S.10). As for the quantity shares, the expenditure shares must sum to one. Furthermore, for two level CES functions, the expenditure shares can be

decomposed into first and second level shares. For example, $\Gamma_Y^U = \Gamma_{\tilde{\mathcal{F}}}^U \Gamma_Y^{\tilde{\mathcal{F}}}$.

Using the specified functional forms, we can derive the following comparative statics conditions based on the first order conditions of producers. In deriving these and the conditions derived from the households utility maximization problem, we use the expressions in (S.16) and (S.17).

Agricultural production (S.18) gives

$$\hat{p}_A - \hat{p}_L - \left(V_A + \frac{1}{\sigma_A}\right)\hat{L}_A + \frac{1}{\sigma_A}\hat{A} = 0, \tag{S.37}$$

$$\hat{p}_A - \hat{p}_P + \frac{1}{\sigma_A}\hat{A} - \left[\left(\frac{1}{\sigma_A} - \frac{1}{\sigma_{\tilde{L}_A}}\right)\Gamma_P^{\tilde{L}_A} + \frac{1}{\sigma_{\tilde{L}_A}}\right]\hat{P}$$
$$-\left(\frac{1}{\sigma_A} - \frac{1}{\sigma_{\tilde{L}_A}}\right)\Gamma_{M_A}^{\tilde{L}_A}\hat{M}_A - \left(\frac{1}{\sigma_A} - \frac{1}{\sigma_{\tilde{L}_A}}\right)\Gamma_W^{\tilde{L}_A}\hat{W} - \left(\frac{1}{\sigma_A} - \frac{1}{\sigma_{\tilde{L}_A}}\right)\Gamma_{\mathcal{E}_A}^{\tilde{L}_A}\hat{\mathcal{E}}_A = 0, \tag{S.38}$$

$$\hat{p}_P - \hat{p}_{M_A} + \frac{1}{\sigma_{\tilde{L}_A}}\hat{P} - \frac{1}{\sigma_{\tilde{L}_A}}\hat{M}_A = 0, \tag{S.39}$$

$$\hat{p}_P - \hat{p}_W + \frac{1}{\sigma_{\tilde{L}_A}}\hat{P} - \frac{1}{\sigma_{\tilde{L}_A}}\hat{W} = 0 \text{ and} \tag{S.40}$$

$$\hat{p}_P - \hat{p}_{\mathcal{E}_A} + \frac{1}{\sigma_{\tilde{L}_A}}\hat{P} - \frac{1}{\sigma_{\tilde{L}_A}}\hat{\mathcal{E}}_A = 0. \tag{S.41}$$

Energy-services production (S.19) gives conditions

$$\hat{p}_{\mathcal{E}} - \hat{p}_E + \frac{1}{\sigma_{\mathcal{E}}}\hat{\mathcal{E}} - \frac{1}{\sigma_{\mathcal{E}}}\hat{E}_{\mathcal{E}} =, \tag{S.42}$$

$$\hat{p}_{\mathcal{E}} - \hat{p}_A + \frac{1}{\sigma_{\mathcal{E}}}\hat{\mathcal{E}} - \frac{1}{\sigma_{\mathcal{E}}}\hat{A}_B = 0 \text{ and} \tag{S.43}$$

$$\hat{p}_{\mathcal{E}} - \hat{p}_R + \frac{1}{\sigma_{\mathcal{E}}}\hat{\mathcal{E}} - \frac{1}{\sigma_{\mathcal{E}}}\hat{R} = 0. \tag{S.44}$$

Fertilizer production (S.20) gives conditions

$$\hat{p}_P - \hat{p}_E + \frac{1}{\sigma_P}\hat{P} - \frac{1}{\sigma_P}\hat{E}_P = 0, \tag{S.45}$$

$$\hat{p}_P - \hat{p}_{\mathcal{P}} + \frac{1}{\sigma_P}\hat{P} - \frac{1}{\sigma_P}\hat{\mathcal{P}} = 0 \text{ and} \tag{S.46}$$

$$\hat{p}_P - \hat{p}_{M_P} + \frac{1}{\sigma_P}\hat{P} - \frac{1}{\sigma_P}\hat{M}_P = 0. \tag{S.47}$$

Timber production (S.21) gives conditions

$$\hat{p}_T - \hat{p}_L + \frac{1}{\sigma_T}\hat{T} - \left(V_T + \frac{1}{\sigma_T}\right)\hat{L}_T = 0 \text{ and} \tag{S.48}$$

$$\hat{p}_T - \hat{p}_{M_T} + \frac{1}{\sigma_T}\hat{T} - \frac{1}{\sigma_T}\hat{M}_T = 0. \tag{S.49}$$

Industrial manufacturing (S.22) gives conditions

$$\hat{p}_Y - \hat{p}_{\mathcal{E}} + \frac{1}{\sigma_Y}\hat{Y} - \frac{1}{\sigma_Y}\hat{\mathcal{E}}_Y = 0 \text{ and} \tag{S.50}$$

$$\hat{p}_Y - \hat{p}_{M_Y} + \frac{1}{\sigma_Y}\hat{Y} - \frac{1}{\sigma_Y}\hat{M}_Y = 0. \tag{S.51}$$

Fisheries (S.23) gives conditions

$$\hat{p}_F - \hat{p}_E + \frac{1}{\sigma_F}\hat{F} - \frac{1}{\sigma_F}\hat{E}_F = 0 \text{ and} \tag{S.52}$$

$$\hat{p}_F - \hat{p}_{M_F} + \frac{1}{\sigma_F}\hat{F} - \frac{1}{\sigma_F}\hat{M}_F = 0. \tag{S.53}$$

For the extraction sectors, fossil fuel extraction (S.24) gives

$$\hat{p}_E - \Lambda_E\hat{E} = \frac{1}{1+\tau_E} \tag{S.54}$$

and the remaining sectors (S.25) give

$$\hat{p}_X - \Lambda_X\hat{X} = 0 \text{ for } X \in \{\mathcal{P}, W, R, M_A, M_F, M_P, M_T, M_Y\}, \tag{S.55}$$

with Λ_X defined in (S.6).

The first order conditions of the households' utility maximization gives conditions

$$\hat{p}_A - \hat{p}_F + \frac{1}{\sigma_{\mathcal{F}}}\hat{A}_{\mathcal{F}} - \frac{1}{\sigma_{\mathcal{F}}}\hat{F} = 0, \quad \text{(S.56)}$$

$$\hat{p}_T - \hat{p}_L + \frac{1}{\sigma_{\tilde{\mathcal{F}}}}\hat{T} - \frac{1}{\sigma_{\tilde{\mathcal{F}}}}\hat{L}_U = 0, \quad \text{(S.57)}$$

$$\hat{p}_T - \hat{p}_Y + \frac{1}{\sigma_{\tilde{\mathcal{F}}}}\hat{T} - \frac{1}{\sigma_{\tilde{\mathcal{F}}}}\hat{Y} = 0 \text{ and} \quad \text{(S.58)}$$

$$\hat{p}_A - \hat{p}_Y + \left[\left(\frac{1}{\sigma_U} - \frac{1}{\sigma_{\mathcal{F}}}\right)\Gamma^{\mathcal{F}}_{A_{\mathcal{F}}} + \frac{1}{\sigma_{\mathcal{F}}}\right]\hat{A}_{\mathcal{F}} + \left(\frac{1}{\sigma_U} - \frac{1}{\sigma_{\mathcal{F}}}\right)\Gamma^{\mathcal{F}}_F\hat{F}$$
$$- \left[\left(\frac{1}{\sigma_U} - \frac{1}{\sigma_{\tilde{\mathcal{F}}}}\right)\Gamma^{\tilde{F}}_Y + \frac{1}{\sigma_{\tilde{\mathcal{F}}}}\right]\hat{Y} - \left(\frac{1}{\sigma_U} - \frac{1}{\sigma_{\tilde{\mathcal{F}}}}\right)\Gamma^{\tilde{\mathcal{F}}}_{L_U}\hat{L}_U - \left(\frac{1}{\sigma_U} - \frac{1}{\sigma_{\tilde{\mathcal{F}}}}\right)\Gamma^{\tilde{\mathcal{F}}}_T\hat{T} = 0. \quad \text{(S.59)}$$

Finally, the budget constraint in (9) gives condition

$$\Gamma^U_{A_{\mathcal{F}}}\hat{p}_A + \Gamma^U_{A_{\mathcal{F}}}\hat{A}_{\mathcal{F}} + \Gamma^U_F\hat{p}_F + \Gamma^U_F\hat{F} + \Gamma^U_Y\hat{p}_Y + \Gamma^U_Y\hat{Y} + \Gamma^U_{L_U}\hat{p}_L + \Gamma^U_{L_U}\hat{L}_U + \Gamma^U_T\hat{p}_T + \Gamma^U_T\hat{T} = 0. \quad \text{(S.60)}$$

Biofuel policy

If we want to include the tax on biofuel (equivalent to a removal of biofuel subsidies), we can replace the first order condition with respect to A_B in (S.19) by

$$p_{\mathcal{E}}\frac{\partial \mathcal{E}}{\partial A_B} - (1 + \tau_B)p_A = 0.$$

This changes comparative statics condition (S.43) to

$$\hat{p}_{\mathcal{E}} - \hat{p}_A + \frac{1}{\sigma_{\mathcal{E}}}\hat{\mathcal{E}} - \frac{1}{\sigma_{\mathcal{E}}}\hat{A}_B = \frac{1}{1 + \tau_B},$$

where the difference is the right hand side.

Sensitivity analysis

There is uncertainty about some of the parameter values. We deal with this by conducting a sensitivity analysis where we test different combinations of values of the uncertain parameters.

These are the values with multiple values given in square brackets in Supplementary Table 4. The choice of parameters to include in the sensitivity analysis was in part based on an analysis of which parameters were the most influential in our model runs. This was determined by calculating the mean relative change of the policy responsiveness of our model variables as a result of increasing (decreasing) each parameter estimates by a fixed percentage. More specifically, we iterated over all parameters of our model, and for each iteration we either increased or decreased each parameter estimate by a fixed percentage (we tried various percentage levels e.g. 5%, 10%, 15% and 20% which all resulted in a similar final ranking). We then ran a simulation using this perturbed parameter estimate and compared the model results to our baseline results by calculating the mean relative change of the policy responsiveness of our model variables as a result of the increase or decrease in a specific parameter estimate. Finally, we ranked all the parameters based on which gave rise to the largest mean relative change of the policy responsiveness of our model variables. The results of this analysis indicated that the results were most sensitive to the elasticity of substitution parameters along with the supply elasticity of fossil fuels.

For these parameters we then ran the model using all possible combinations of the minimum and maximum values of these parameters (with the remaining parameters at their baseline values). For each model variable we then find the minimum and maximum values of the change induced by the policy in the different runs. These values are reported in Table 4 for the carbon tax with and without the accompanying biofuel policy.

Supplementary Tables

Supplementary Table 1: **Earth system processes and their drivers.** The table lists the main pressures on critical ESPs and the economic activities driving them (with percent of total pressure in parenthesis).

ESP	Main pressure	Principal economic drivers
Climate change	CO_2 and other greenhouse gas emissions	Energy prod. (67%), agriculture (14%), land-use change (12%)
Biogeochemical flows	Phosphorus and Nitrogen emissions	Fertilizer use in agriculture (90%)
Ocean acidification	Increased H+ concentration caused by CO_2 emissions	Same as climate change
Freshwater use	Over consumption	Freshwater use in agriculture (92%).
Land-system change	Forest-land loss	Deforestation due to agricultural expansion (96%)
Biodiversity loss	Extinction of species	Agriculture threatens 62%, logging 46%, climate change 19% and fishing 13% of red listed species.†
Stratospheric ozone depletion	Emissions of ozone-depleting substances	Largely resolved, remaining depletion mainly caused by NO_2 emissions from agriculture, fossil fuels, manufacturing and biofuels.
Aerosol loading	Emissions of black and organic carbon, sulfates, nitrates	Fossil and biofuel consumption (95%), biomass burning (5%)†
Chemical pollution	Emissions of non-natural chemicals	Manufacturing, fossil fuels, agriculture and fertilizers.

† Biodiversity loss percentages indicate share of total threatened species by activity. Note that any single species may be subject to more than one threat (hence percentages can sum to more than 100%). Percentage shares of aerosol optical depth contributions for black and organic carbon and sulfur derived from [22] and [21] (see Methods).

Supplementary Table 2: **Effects on ESPs – carbon policy.** Change in planetary pressure from a global carbon tax of 1%. Shows the percentage change to our pressure variables for each respective Earth system process resulting from the perturbation of the model variables following the tax. Land-use refers to change in natural land. Ozone depletion and chemical pollution are only assessed qualitatively (-1 denotes a reduction).

	Climate	*Biodiv.*	*Biochem.*	*Freshw.*	*Ocean acid.*	*Land-use*	*Aerosols*	*Ozone*	*Chem.*
Land-Share Agriculture (L_A)	0.0	0.0	0.0	0.0	0.0	0.0	0.0	0	0
Land-Share Timber (L_T)	0.0	0.0	0.0	0.0	0.0	0.0	0.0	0	0
Land-Share Other (L_U)	0.0017	0.0	0.0	0.0	0.0017	-0.0143	0.0	0	0
Fossil-Fuel Extracted (E)	-0.0328	-0.0008	0.0	0.0	-0.0328	0.0	0.0	-1	-1
Fossil-Fuel Use Energy Serv. ($E_{\mathcal{E}}$)	-0.2162	0.0	0.0	0.0	-0.2162	0.0	-0.0138	0	0
Fossil-Fuel Use Fertilizer Prod. (E_P)	-0.0018	0.0	-0.1349	0.0	-0.0018	0.0	-0.0001	0	0
Fossil-Fuel Use Fisheries (E_F)	-0.0007	0.0	0.0	0.0	-0.0007	0.0	-0.0	0	0
Agriculture Total Production (A)	-0.0004	-0.0007	0.0	0.0	-0.0004	0.0	0.0	-1	-1
Agriculture Prod. For Biofuels (A_B)	0.0	0.0	0.0	0.0	0.0	0.0	0.0002	1	0
Agriculture Prod. For Food (A_F)	0.0	0.0	0.0	0.0	0.0	0.0	0.0	0	0
Energy Services ($\mathcal{E}$)	0.0	0.0	0.0	0.0	0.0	0.0	0.0	0	0
Energy For Agriculture ($\mathcal{E}_A$)	0.0	0.0	0.0	0.0	0.0	0.0	0.0	0	0
Energy Services For Final Goods ($\mathcal{E}_Y$)	0.0	0.0	0.0	0.0	0.0	0.0	0.0	0	0
Fertilizer Production (P)	0.0	-0.0012	0.0	0.0	0.0	0.0	0.0	0	0
Water Production (W)	0.0	0.0	0.0	0.0091	0.0	0.0	0.0	0	0
Phosphate Extraction ($\mathcal{P}$)	0.0	0.0	-0.0068	0.0	0.0	0.0	0.0	0	0
Renewables Production (R)	0.0	0.001	0.0	0.0	0.0	0.0	0.0	0	0
Fisheries Production (F)	0.0	-0.0044	0.0	0.0	0.0	0.0	0.0	0	0
Timber Production (T)	0.0	0.0005	0.0	0.0	0.0	0.0	0.0	0	0
Final Goods (Y)	-0.0013	-0.0042	0.0	0.0	-0.0013	0.0	0.0	-1	-1
Intermediaries Agriculture. (M_A)	0.0	0.0	0.0	0.0	0.0	0.0	0.0	0	0
Intermediaries Timber. (M_T)	0.0	0.0	0.0	0.0	0.0	0.0	0.0	0	0
Intermediaries Fisheries (M_F)	0.0	0.0	0.0	0.0	0.0	0.0	0.0	0	0
Intermediaries Final Goods. (M_Y)	0.0	0.0	0.0	0.0	0.0	0.0	0.0	0	0
Intermediaries Fertilizers (M_P)	0.0	0.0	0.0	0.0	0.0	0.0	0.0	0	0
Total impact	-0.2514	-0.0098	-0.1417	0.0091	-0.2514	-0.0143	-0.0136	-1	0

Supplementary Table 3: **Effects on ESPs – carbon and biofuel policy.** Change in planetary pressure from a global carbon and biofuel tax of 1%. Shows the percentage change to our pressure variables for each respective Earth system process resulting from the perturbation of the model variables following the tax. Land-use refers to change in natural land. Ozone depletion and chemical pollution are only assessed qualitatively (-1 denotes a reduction).

	Climate	*Biodiv.*	*Biochem.*	*Freshw.*	*Ocean acid.*	*Land-use*	*Aerosols*	*Ozone*	*Chem.*
Land-Share Agriculture (L_A)	0.0	0.0	0.0	0.0	0.0	0.0	0.0	0	0
Land-Share Timber (L_T)	0.0	0.0	0.0	0.0	0.0	0.0	0.0	0	0
Land-Share Other (L_U)	-0.0053	0.0	0.0	0.0	-0.0053	0.0431	-0.0001	0	0
Fossil-Fuel Extracted (E)	-0.0324	-0.0008	0.0	0.0	-0.0324	0.0	0.0	-1	-1
Fossil-Fuel Use Energy Serv. ($E_{\mathcal{E}}$)	-0.2128	0.0	0.0	0.0	-0.2128	0.0	-0.0135	0	0
Fossil-Fuel Use Fertilizer Prod. (E_P)	-0.0024	0.0	-0.1841	0.0	-0.0024	0.0	-0.0001	0	0
Fossil-Fuel Use Fisheries (E_F)	-0.0007	0.0	0.0	0.0	-0.0007	0.0	-0.0	0	0
Agriculture Total Production (A)	-0.0062	-0.0092	0.0	0.0	-0.0062	0.0	0.0	-1	-1
Agriculture Prod. For Biofuels (A_B)	0.0	0.0	0.0	0.0	0.0	0.0	-0.0003	-1	0
Agriculture Prod. For Food (A_F)	0.0	0.0	0.0	0.0	0.0	0.0	0.0	0	0
Energy Services ($\mathcal{E}$)	0.0	0.0	0.0	0.0	0.0	0.0	0.0	0	0
Energy For Agriculture ($\mathcal{E}_A$)	0.0	0.0	0.0	0.0	0.0	0.0	0.0	0	0
Energy Services For Final Goods ($\mathcal{E}_Y$)	0.0	0.0	0.0	0.0	0.0	0.0	0.0	0	0
Fertilizer Production (P)	0.0	-0.0037	0.0	0.0	0.0	0.0	0.0	0	0
Water Production (W)	0.0	0.0	0.0	-0.0359	0.0	0.0	0.0	0	0
Phosphate Extraction ($\mathcal{P}$)	0.0	0.0	-0.0493	0.0	0.0	0.0	0.0	0	0
Renewables Production (R)	0.0	0.001	0.0	0.0	0.0	0.0	0.0	0	0
Fisheries Production (F)	0.0	-0.0048	0.0	0.0	0.0	0.0	0.0	0	0
Timber Production (T)	0.0	0.0029	0.0	0.0	0.0	0.0	0.0	0	0
Final Goods (Y)	-0.0011	-0.0036	0.0	0.0	-0.0011	0.0	0.0	-1	-1
Intermediaries Agriculture. (M_A)	0.0	0.0	0.0	0.0	0.0	0.0	0.0	0	0
Intermediaries Timber. (M_T)	0.0	0.0	0.0	0.0	0.0	0.0	0.0	0	0
Intermediaries Fisheries (M_F)	0.0	0.0	0.0	0.0	0.0	0.0	0.0	0	0
Intermediaries Final Goods. (M_Y)	0.0	0.0	0.0	0.0	0.0	0.0	0.0	0	0
Intermediaries Fertilizers (M_P)	0.0	0.0	0.0	0.0	0.0	0.0	0.0	0	0
Total impact	-0.2609	-0.0183	-0.2333	-0.0359	-0.2609	0.0431	-0.0141	-1	-1

Supplementary Table 4: **Sensitivity analysis.** Minimum and maximum changes (over different parameter combinations - see Table 4) for the model variables in response to: (i) a one-percent increase in the carbon tax; (ii) a one-percent increase in the carbon tax plus a one-percent reduction of biofuel subsidies.

	Carbon tax		*Carbon Tax + Biofuel policy*	
Variable	*Min*	*Max*	*Min*	*Max*
Agricultural Sector: Production				
Total	-0.0173	0.0303	-0.0813	-0.0229
Biofuels	0.19	1.5561	-1.6504	-0.3552
Food	-0.06	-0.0157	-0.0395	0.0211
Agricultural Sector: Inputs				
Land-Share Agriculture	-0.0111	0.057	-0.0731	-0.0002
Energy in Agriculture	-0.5854	-0.1067	-0.6476	-0.1476
Fertilizer Production	-0.0427	0.0139	-0.1047	-0.0352
Water Production	-0.0115	0.0409	-0.0729	-0.0128
Energy-related sectors and services				
Fossil-Fuel in Energy Services	-0.5709	-0.1514	-0.5691	-0.1465
Fossil-Fuel in Fertilizer Prod.	-0.2676	-0.0227	-0.3246	-0.0604
Fossil-Fuel in Fisheries	-0.9154	-0.0888	-0.9354	-0.0906
Energy Services	-0.5209	-0.101	-0.5263	-0.1017
Energy in Manufacturing	-0.533	-0.0956	-0.5356	-0.094
Renewables Production	0.1424	0.9226	0.1421	0.9322
Extractive Sectors				
Fossil-Fuel Extraction	-0.564	-0.1507	-0.5632	-0.146
Phosphate Extraction	-0.0365	0.0363	-0.0963	-0.0158
Other				
Land-Share Timber	-0.0483	0.0319	0.0003	0.0749
Land-Share Natural	-0.066	0.0122	0.0001	0.084
Fisheries Production	-0.2475	-0.0485	-0.2703	-0.05
Timber Production	-0.0314	0.035	0.0005	0.0749
Manufacturing	-0.0562	-0.0215	-0.0507	-0.0109

Supplementary References

[1] IPCC. *Climate Change 2013: The Physical Science Basis. Contribution of Working Group I to the Fifth Assessment Report of the Intergovernmental Panel on Climate Change* (Cambridge University Press, Cambridge, United Kingdom and New York, NY, USA, 2013). Available at: `www.climatechange2013.org`.

[2] Steffen, W. *et al.* Planetary boundaries: Guiding human development on a changing planet. *Science* **347**, 1259855 (2015).

[3] Herzog, T. World Greenhouse Gas Emissions in 2005, World Resources Institute (2005).

[4] Prudhomme, M. World phosphate rock flows, losses and uses. In *International Fertilizer Industry Association, Phosphates 2010 International Conference*, 22–24 (2010).

[5] Howarth, R. W. Coastal nitrogen pollution: a review of sources and trends globally and regionally. *Harmful Algae* **8**, 14–20 (2008).

[6] Byerlee, D., Stevenson, J. & Villoria, N. Does intensification slow crop land expansion or encourage deforestation? *Global Food Security* **3**, 92–98 (2014).

[7] Bouwman, L. *et al.* Exploring global changes in nitrogen and phosphorus cycles in agriculture induced by livestock production over the 1900–2050 period. *Proceedings of the National Academy of Sciences* **110**, 20882–20887 (2013).

[8] Huang, W.-y. *Impact of rising natural gas prices on US ammonia supply* (DIANE Publishing, 2007).

[9] Sunda, W. G. & Cai, W.-J. Eutrophication induced co2-acidification of subsurface coastal waters: Interactive effects of temperature, salinity, and atmospheric p co2. *Environmental science & technology* **46**, 10651–10659 (2012).

[10] Hoekstra, A. Y. & Mekonnen, M. M. The water footprint of humanity. *Proceedings of the national academy of sciences* **109**, 3232–3237 (2012).

[11] Foley, J. A. *et al.* Solutions for a cultivated planet. *Nature* **478**, 337–342 (2011).

[12] Foley, J. A. *et al.* Global consequences of land use. *Science* **309**, 570–574 (2005).

[13] Gibbs, H. K. *et al.* Tropical forests were the primary sources of new agricultural land in the 1980s and 1990s. *Proceedings of the National Academy of Sciences* **107**, 16732–16737 (2010).

[14] Lambin, E. F. & Geist, H. J. Proximate Causes and Underlying Driving Forces of Tropical Deforestation: Tropical forests are disappearing as the result of many pressures, both local and regional, acting in various combinations in different geographical locations. *BioScience* **52**, 143–150 (2002).

[15] Maxwell, S. L., Fuller, R. A., Brooks, T. M. & Watson, J. E. Biodiversity: The ravages of guns, nets and bulldozers. *Nature* **536**, 143–145 (2016).

[16] Worm, B. *et al.* Impacts of biodiversity loss on ocean ecosystem services. *Science* **314**, 787–790 (2006). Available at: `http://science.sciencemag.org/content/314/5800/787`.

[17] Rice, J. C. & Garcia, S. M. Fisheries, food security, climate change, and biodiversity: characteristics of the sector and perspectives on emerging issues. *ICES Journal of Marine Science* **68**, 1343–1353 (2011).

[18] Halpern, B. S. *et al.* A Global Map of Human Impact on Marine Ecosystems. *Science* **319**, 948–952 (2008). Available at: `http://science.sciencemag.org/content/319/5865/948`.

[19] Boucher, O. *et al.* Clouds and aerosols. In *Climate change 2013: the physical science basis. Contribution of Working Group I to the Fifth Assessment Report of the Intergovernmental Panel on Climate Change*, 571–657 (Cambridge University Press, 2013).

[20] Rockström, J. *et al.* Planetary boundaries: exploring the safe operating space for humanity. *Ecology and society* **14** (2009).

[21] Streets, D. G. *et al.* Anthropogenic and natural contributions to regional trends in aerosol optical depth, 1980–2006. *Journal of Geophysical Research: Atmospheres* **114** (2009).

[22] Lamarque, J.-F. *et al.* Historical (1850–2000) gridded anthropogenic and biomass burning emissions of reactive gases and aerosols: methodology and application. *Atmospheric Chemistry and Physics* **10**, 7017–7039 (2010).

[23] Ravishankara, A., Daniel, J. S. & Portmann, R. W. Nitrous oxide (n2o): the dominant ozone-depleting substance emitted in the 21st century. *science* **326**, 123–125 (2009).

[24] Solomon, S. *et al.* *Climate change 2007-the physical science basis: Working group I contribution to the fourth assessment report of the IPCC*, vol. 4 (Cambridge university press, 2007).

[25] Andersen, S. O., Metz, B., Kuijpers, L. & Solomon, S. *Safeguarding the Ozone Layer and the Global Climate System: Special Report of the Intergovernmental Panel on Climate Change* (Cambridge University Press, 2005).

[26] Rockström, J. *et al.* A safe operating space for humanity. *Nature* **461**, 472–475 (2009).

[27] Zeng, Y., Tian, S. & Pan, Y. Revealing the sources of atmospheric ammonia: a review. *Current Pollution Reports* **4**, 189–197 (2018).

[28] Division, S. *FAO Statistical Pocketbook* (Food and Agriculture Organization of the United Nations Rome, 2015).

[29] Campbell, B. M. *et al.* Agriculture production as a major driver of the earth system exceeding planetary boundaries. *Ecology and Society* **22** (2017).

[30] Golosov, M., Hassler, J., Krusell, P. & Tsyvinski, A. Optimal taxes on fossil fuel in general equilibrium. *Econometrica* **82**, 41–88 (2014).

[31] Metcalf, G. E. On the economics of a carbon tax for the United States. *Brookings Papers on Economic Activity* **2019**, 405–484 (2019).

[32] Barron, A. R., Fawcett, A. A., Hafstead, M. A., McFarland, J. R. & Morris, A. C. Policy insights from the EMF 32 study on US carbon tax scenarios. *Climate change economics* **9**, 1840003 (2018).

[33] Andersson, J. J. Carbon Taxes and CO2 Emissions: Sweden as a Case Study. *American Economic Journal: Economic Policy* **11**, 1–30 (2019).
